# Supplementary material for: Quantitative Health Risk Assessment of the Chronic Inhalation of Chemical Compounds in Healthcare and Elderly Care Facilities
Source: Toxics. 2022 Mar 15;10(3):141. doi: 10.3390/toxics10030141 (PMC8954219; doi:10.3390/toxics10030141)
Supplement: Supplementary file 1 [file toxics-10-00141-s001.zip › toxics-1604645-supplementary.pdf]

Supplementary Materials: Quantitative Health Risk Assessment of the Chronic Inhalation of Chemical Compounds in Healthcare and Elderly Care Facilities

Anaïs Colas, Alexandre Baudet, Pierre Le Cann, Olivier Blanchard, Jean-Pierre Gangneux, Estelle Baurès, and Arnaud Florentin

Table S1. Compounds of interest's toxicity from REACH regulation (inhalation) and selected VTR.

Organic compound	Acute toxicity	Carcinogenicity	Specific target organ toxicity	Selected TRVs (threshold/non-threshold effects)
**VOC**				
benzene	data conclusive but not sufficient for classification	Carc. 1A	hematopoietic system	10 µg/m^3 (ANSES)/2.6E-05 (µg/m^3)$^{-1}$ (ANSES)
ethylbenzene	Acute Tox. 4	data conclusive but not sufficient for classification	hearing organs	1.5 µg/m^3 (ANSES)/2.5E-06 (µg/m^3)$^{-1}$ (OEHHA)
styrene	Acute Tox. 4	data conclusive but not sufficient for classification	nose, ear	860 µg/m^3 (ATSDR)/ -
toluene	data conclusive but not sufficient for classification	-	neurologic	19000 µg/m^3 (ANSES)/ -
o-xylene	Acute Tox. 4	data conclusive but not sufficient for classification	respiratory tract	- / -
mp-xylenes	Acute Tox. 4	data conclusive but not sufficient for classification	respiratory tract	- / -
1,2,4-trimethylbenzene	Acute Tox. 4	data conclusive but not sufficient for classification	respiratory tract	60 µg/m^3 (US EPA)/ -
naphthalene	data conclusive but not sufficient for classification	Carc. 2	data conclusive but not sufficient for classification	37 µg/m^3 (ANSES)/5.6E-06 (µg/m^3)$^{-1}$ (ANSES)
phenol	Acute Tox. 3	data conclusive but not sufficient for classification	kidney, liver, skin, nervous system	20 µg/m^3 (RIVM)/ -
n-decane	data conclusive but not sufficient for classification	data conclusive but not sufficient for classification	data conclusive but not sufficient for classification	- / -
n-undecane	data conclusive but not sufficient for classification	data conclusive but not sufficient for classification	data conclusive but not sufficient for classification	- / -
n-heptane	data conclusive but not sufficient for classification	data conclusive but not sufficient for classification	central nervous system	18400 µg/m^3 (RIVM)/ -
1,1,1-trichloroethane	Acute Tox. 4	data conclusive but not sufficient for classification	data conclusive but not sufficient for classification	5000 µg/m^3 (OEHHA)/ -

1,4-dichlorobenzene	data conclusive but not sufficient for classification	Carc. 2	data conclusive but not sufficient for classification	60 µg/m^3 (ATSDR)/1.1E-05 (µg/m^3)$^{-1}$ (OEHHA)
trichloroethylene	data conclusive but not sufficient for classification	Carc. 1B	central nervous system	2.2 µg/m^3 (ATSDR)/1.0E-06 (µg/m^3)$^{-1}$ (ANSES)
tetrachloroethylene	data conclusive but not sufficient for classification	Carc. 2	data conclusive but not sufficient for classification	400 µg/m^3 (ANSES)/2.6E-07 (µg/m^3)$^{-1}$ (ANSES)
bromodichloromethane	-	-	-	- / -
tribromomethane	Acute Tox. 3	data conclusive but not sufficient for classification	data conclusive but not sufficient for classification	- /2.3E-05 (µg/m^3)$^{-1}$ (US EPA)
trichloromethane	Acute Tox. 4	Carc. 2	central nervous system, liver, kidneys	63 µg/m^3 (ANSES)/2.3E-05 (µg/m^3)$^{-1}$ (US EPA)
2-ethyl-1-hexanol	Acute Tox. 4	data conclusive but not sufficient for classification	respiratory tract	4 µg/m^3 (US EPA)/ -
ethanol	data conclusive but not sufficient for classification	data conclusive but not sufficient for classification	data conclusive but not sufficient for classification	- / -
isopropanol	data conclusive but not sufficient for classification	data conclusive but not sufficient for classification	may cause drowsiness or dizziness	7000 µg/m^3 (OEHHA)/ -
n-propanol	data conclusive but not sufficient for classification	-	may cause drowsiness or dizziness	- / -
acetone	data conclusive but not sufficient for classification	data conclusive but not sufficient for classification	narcotic effects	30 µg/m^3 (ATSDR)/ -
2-butanone	data conclusive but not sufficient for classification	-	central nervous system (narcotic effects)	5000 µg/m^3 (US EPA)/ -
limonene	data conclusive but not sufficient for classification	data conclusive but not sufficient for classification	data conclusive but not sufficient for classification	- / -
ether	data conclusive but not sufficient for classification	-	may cause drowsiness or dizziness	- / -
2-ethoxyethanol	Acute Tox. 3	data conclusive but not sufficient for classification	data conclusive but not sufficient for classification	70 µg/m^3 (ANSES)/ -
2-butoxyethanol	data conclusive but not sufficient for classification	data conclusive but not sufficient for classification	data conclusive but not sufficient for classification	1600 µg/m^3 (US EPA)/ -
formaldehyde	Acute Tox. 3	Carc. 1B	data conclusive but not sufficient for classification	123 µg/m^3 (ANSES)/1.3E-05 (µg/m^3)$^{-1}$ (US EPA)
acetaldehyde	data conclusive but not sufficient for classification	Carc. 2	respiratory tract	9 µg/m^3 (USEPA)/2.2E-06 (µg/m^3)$^{-1}$ (US EPA)
propionaldehyde	data conclusive but not sufficient for classification	data conclusive but not sufficient for classification	respiratory system	63 µg/m^3 (ANSES)/2.3E-05 (µg/m^3)$^{-1}$ (US EPA)

butyraldehyde	data conclusive but not sufficient for classification	data conclusive but not sufficient for classification	data conclusive but not sufficient for classification	8 μg/m³ (US EPA)/ -
isovaleraldehyde	data conclusive but not sufficient for classification	data conclusive but not sufficient for classification	may cause respiratory irritation.	- / -
valeraldehyde	data conclusive but not sufficient for classification	data conclusive but not sufficient for classification	may cause respiratory irritation.	- / -
hexaldehyde	-	-	-	- / -
**SVOC**				
di(2-ethylhexyl) phthalate (DEHP)	data conclusive but not sufficient for classification	data conclusive but not sufficient for classification	data conclusive but not sufficient for classification	- /2.4E-06 (μg/m³)$^{-1}$ (OEHHA)
diethylphthalate (DEP)	data conclusive but not sufficient for classification	data conclusive but not sufficient for classification	data conclusive but not sufficient for classification	- / -
dibutylphthalate (DBP)	data conclusive but not sufficient for classification	data conclusive but not sufficient for classification	data conclusive but not sufficient for classification	- / -
diisobutylt-phthalate (DiBP)	data conclusive but not sufficient for classification	data conclusive but not sufficient for classification	data conclusive but not sufficient for classification	- / -
benzyl-butylphthalate (BBP)	data conclusive but not sufficient for classification	data conclusive but not sufficient for classification	data conclusive but not sufficient for classification	- / -
diisononylphthalate (DiNP)	data conclusive but not sufficient for classification	data conclusive but not sufficient for classification	data conclusive but not sufficient for classification	- / -
tonalide	data conclusive but not sufficient for classification	data conclusive but not sufficient for classification	data conclusive but not sufficient for classification	- / -
galaxolide	data conclusive but not sufficient for classification	data conclusive but not sufficient for classification	data conclusive but not sufficient for classification	- / -
cyfluthrine	-	-	-	- / -
cypermethrine	-	-	-	- / -
deltamethrine	-	-	-	- / -
permethrine	-	-	-	- / -
tetramethrine	-	-	-	- / -

Notes: “-“ indicates a data lacking

Table S2. Hazard quotients in healthcare and elderly care facilities.

	**Dental and general practitioner offices**			**Nursing homes**				**Pharmacies**		**Hospitals**			
	Dental surgeon	Dental assistant	General practitioner	Nursing home’ s nurse	Nursing home’ s nurse assistant	Nursing home’ s physiotherapist	Nursing home’ s resident	Pharmacist	Pharmacy technician	Endoscope Disinfection Unit’ s	Laboratory technician	Care unit’ s nurse	Anesthesia Care Unit’ s nurse
Aromatic hydrocarbons													
Benzene	1.22E-02	1.22E-02	1.19E-02	1.13E-02	1.46E-02	1.28E-02	3.30E-03	8.76E-03	9.55E-03	5.07E-03	5.04E-03	6.44E-03	5.36E-03
Ethylbenzene	2.41E-01	1.92E-01	1.53E-02	2.85E-02	4.37E-02	3.02E-02	1.05E-02	4.46E-02	4.57E-02	1.17E-02	5.53E-02	8.84E-03	1.12E-02
Styrene	6.83E-05	6.08E-05	2.13E-05	2.19E-05	3.05E-05	2.41E-05	7.09E-06	8.79E-05	9.54E-05	6.65E-06	1.81E-05	1.37E-05	1.39E-05

Toluene	5.90E-06	5.69E-06	5.31E-06	5.66E-06	7.19E-06	6.39E-06	1.61E-06	9.95E-06	1.08E-05	5.18E-06	2.15E-05	4.26E-06	5.89E-06
o-xylene	1.84E-06	1.51E-06	4.91E-07	7.30E-07	1.20E-06	7.54E-07	2.97E-07	7.13E-07	7.80E-07	1.26E-07	1.63E-06	2.15E-07	2.91E-07
mp-xylenes	4.63E-06	3.80E-06	9.12E-07	1.63E-06	2.76E-06	1.66E-06	6.86E-07	1.70E-06	1.84E-06	5.39E-07	4.63E-06	4.93E-07	6.78E-07
1.2.4-trimethylbenzene	6.77E-04	6.37E-04	5.35E-04	3.42E-04	5.10E-04	3.67E-04	1.22E-04	5.84E-04	6.61E-04	0.00E+00	0.00E+00	0.00E+00	0.00E+00
Naphthalene	5.98E-04	6.00E-04	4.95E-04	4.65E-04	5.91E-04	5.25E-04	1.32E-04	3.49E-04	3.74E-04	1.28E-04	2.22E-04	1.99E-04	2.17E-04
Phenol	4.50E-03	4.31E-03	2.98E-03	2.83E-03	3.93E-03	3.11E-03	9.14E-04	2.86E-03	3.09E-03	6.04E-04	1.47E-03	1.37E-03	1.19E-03
Aliphatic hydrocarbons													
n-decane	NC	NC	NC	NC	NC	NC	NC	NC	NC	NC	NC	NC	NC
n-heptane	1.34E-06	1.32E-06	9.96E-07	1.26E-06	1.72E-06	1.39E-06	3.98E-07	1.35E-06	1.48E-06	2.82E-07	6.30E-07	3.09E-07	5.14E-07
n-undecane	NC	NC	NC	NC	NC	NC	NC	NC	NC	NC	NC	NC	NC
Halogenated hydrocarbons													
1.1.1-Trichloroethane	9.92E-06	9.87E-06	1.10E-05	9.99E-06	1.22E-05	1.14E-05	2.70E-06	6.92E-06	7.52E-06	0.00E+00	0.00E+00	0.00E+00	0.00E+00
1.4-Dichlorobenzene	3.24E-04	3.32E-04	3.05E-04	2.87E-04	3.64E-04	3.24E-04	8.16E-05	2.15E-04	2.31E-04	0.00E+00	0.00E+00	0.00E+00	0.00E+00
Trichloroethylene	8.85E-03	9.06E-03	8.33E-03	7.82E-03	9.94E-03	8.83E-03	2.23E-03	5.87E-03	6.29E-03	2.16E-03	3.74E-03	3.35E-03	3.65E-03
Tetrachloroethylene	2.18E-04	2.04E-04	4.58E-05	4.30E-05	5.46E-05	4.86E-05	1.22E-05	2.46E-04	2.84E-04	1.19E-05	1.17E-05	1.84E-05	2.01E-05
Trichloromethane	4.00E-04	4.19E-04	2.91E-04	2.73E-04	3.47E-04	3.08E-04	7.77E-05	2.42E-04	2.67E-04	7.54E-05	4.80E-04	1.23E-04	1.27E-04
Dibromochloromethane	NC	NC	NC	NC	NC	NC	NC	NC	NC	NC	NC	NC	NC
Tribromomethane	NC	NC	NC	NC	NC	NC	NC	NC	NC	NC	NC	NC	NC
Alcohols													
2-ethyl-1-hexanol	1.87E-01	1.78E-01	1.23E-01	1.14E-01	1.45E-01	1.29E-01	3.24E-02	1.02E-01	1.08E-01	3.04E-02	5.07E-02	4.69E-02	5.11E-02
Ethanol	3.10E-04	3.32E-04	2.81E-04	2.80E-04	4.55E-04	2.90E-04	1.12E-04	2.06E-04	2.06E-04	4.94E-05	1.81E-04	2.09E-04	2.24E-04
Isopropanol	5.32E-04	5.66E-04	5.32E-04	1.87E-04	2.86E-04	1.99E-04	6.87E-05	2.05E-04	2.17E-04	4.40E-05	2.05E-04	2.66E-04	1.90E-04
n-propanol	2.54E-05	2.29E-05	1.26E-06	1.18E-06	1.50E-06	1.34E-06	3.37E-07	1.54E-06	1.62E-06	3.02E-07	1.63E-06	1.01E-06	6.07E-07
Ketones													
Acetone	1.16E-01	1.27E-01	9.19E-02	4.44E-02	6.67E-02	4.75E-02	1.60E-02	4.52E-02	5.00E-02	1.56E-02	5.28E-02	3.33E-02	3.00E-02
2-Butanone	6.14E-05	7.08E-05	2.02E-05	1.89E-05	2.40E-05	2.14E-05	5.39E-06	1.52E-05	1.68E-05	0.00E+00	0.00E+00	0.00E+00	0.00E+00
Terpenes													
Limonene	NC	NC	NC	NC	NC	NC	NC	NC	NC	NC	NC	NC	NC
Ethers													
Ether	9.19E-07	9.42E-07	1.01E-06	9.16E-07	1.12E-06	1.05E-06	2.48E-07	3.42E-06	2.59E-06	1.98E-06	3.09E-05	2.14E-06	7.36E-06
2-Ethoxyethanol	6.87E-03	6.90E-03	7.00E-03	6.51E-03	8.27E-03	7.35E-03	1.85E-03	4.97E-03	5.41E-03	0.00E+00	0.00E+00	0.00E+00	0.00E+00
2-Butoxyethanol	3.01E-04	3.02E-04	3.06E-04	2.88E-04	3.70E-04	3.24E-04	8.33E-05	2.18E-04	2.37E-04	0.00E+00	0.00E+00	0.00E+00	0.00E+00
Aldehydes													
Formaldehyde	7.16E-03	7.07E-03	2.34E-02	7.30E-03	1.19E-02	7.58E-03	2.92E-03	1.04E-02	1.09E-02	1.05E-03	2.76E-03	1.78E-03	2.21E-03
Acetaldehyde	7.46E-02	7.27E-02	6.52E-02	5.18E-02	7.05E-02	5.72E-02	1.63E-02	6.79E-02	7.17E-02	1.00E-02	4.33E-02	2.58E-02	2.53E-02
Propionaldehyde	8.06E-03	8.22E-03	1.20E-02	7.73E-03	1.01E-02	8.66E-03	2.29E-03	1.20E-02	1.27E-02	6.31E-03	1.03E-02	9.67E-03	1.03E-02
Butyraldehyde	NC	NC	NC	NC	NC	NC	NC	NC	NC	NC	NC	NC	NC
Isovaleraldehyde	NC	NC	NC	NC	NC	NC	NC	NC	NC	NC	NC	NC	NC
Valeraldehyde	NC	NC	NC	NC	NC	NC	NC	NC	NC	NC	NC	NC	NC
Hexaldehyde	4.10E-05	4.08E-05	1.06E-04	5.27E-05	7.01E-05	5.87E-05	1.60E-05	1.22E-04	1.29E-04	1.74E-05	3.17E-05	3.01E-05	3.00E-05
Phthalates													

di(2-ethyl hexyl)phthalate (DEHP)	NC	NC	NC	NC	NC	NC	NC	NC	NC	NC	NC	NC	NC
Diethylphthalate (DEP)	4.53E-06	4.58E-06	2.08E-05	1.33E-05	2.34E-05	1.34E-05	5.88E-06	5.56E-06	6.29E-06	6.64E-07	2.96E-06	2.68E-06	3.04E-06
Dibutylphthalate (DBP)	2.02E-04	2.01E-04	5.78E-04	3.58E-04	5.50E-04	3.79E-04	1.33E-04	2.17E-04	2.47E-04	1.30E-04	2.43E-04	1.62E-04	1.52E-04
Diisobutylphthalate (DiBP)	3.92E-05	3.93E-05	3.98E-05	4.02E-05	6.52E-05	4.18E-05	1.60E-05	2.18E-05	2.34E-05	1.50E-05	2.44E-05	4.24E-05	1.48E-05
Benzylbutylphthalate (BBP)	1.22E-06	1.38E-06	8.93E-06	1.90E-06	2.42E-06	2.15E-06	5.41E-07	1.37E-06	1.48E-06	5.53E-07	9.13E-07	8.52E-07	9.13E-07
Diisononylphthalate (DiNP)	1.31E-07	1.46E-07	2.83E-07	1.17E-07	1.65E-07	1.28E-07	3.86E-08	7.16E-08	7.62E-08	2.82E-08	4.66E-08	4.35E-08	4.66E-08
Musks													
Tonalide (AHTN)	3.17E-05	3.56E-05	5.50E-05	4.44E-05	6.16E-05	4.88E-05	1.43E-05	1.24E-04	1.52E-04	0.00E+00	2.56E-05	2.29E-05	9.04E-06
Galaxolide (HHCB)	1.76E-06	1.64E-06	8.36E-06	2.91E-06	4.17E-06	3.17E-06	9.81E-07	5.31E-06	6.32E-06	2.16E-07	1.65E-06	1.52E-06	9.71E-07
Pyrethrinoides													
Cyfluthrine	NC	NC	NC	NC	NC	NC	NC	NC	NC	NC	NC	NC	NC
Cypermethrine	NC	NC	NC	NC	NC	NC	NC	NC	NC	NC	NC	NC	NC
Deltamethrine	NC	NC	NC	NC	NC	NC	NC	NC	NC	NC	NC	NC	NC
Permethrine	NC	NC	NC	NC	NC	NC	NC	NC	NC	NC	NC	NC	NC
Tetramethrine	NC	NC	NC	NC	NC	NC	NC	NC	NC	NC	NC	NC	NC

Notes: Orange values indicates hazard quotients greater than 0.1; NC indicates that the hazard quotient was not calculated due to the absence of TRV and DNEL for chronic inhalation.

Table S3. Excess lifetime cancer risk in healthcare and elderly care facilities.

	Dental and general practitioner offices			Nursing homes				Pharmacies		Hospitals			
	Dental surgeon	Dental assistant	General practitioner	Nursing home's nurse	Nursing home's nurse assistant	Nursing home's physiotherapist	Nursing home's resident	Pharmacist	Pharmacy technician	Endoscope Disinfection Unit's	Laboratory technician	Care unit's nurse	Anesthesia Care Unit's nurse
Aromatic hydrocarbons													
Benzene	3,16E-06	3,18E-06	3,10E-06	2,95E-06	3,81E-06	3,32E-06	8,59E-07	2,28E-06	2,48E-06	1,32E-06	1,31E-06	1,67E-06	1,39E-06
Ethylbenzene	9,05E-07	7,20E-07	5,73E-08	1,07E-07	1,64E-07	1,13E-07	3,95E-08	1,67E-07	1,71E-07	4,37E-08	2,07E-07	3,31E-08	4,19E-08
Styrene	NC	NC	NC	NC	NC	NC	NC	NC	NC	NC	NC	NC	NC
Toluene	NC	NC	NC	NC	NC	NC	NC	NC	NC	NC	NC	NC	NC
o-xylene	NC	NC	NC	NC	NC	NC	NC	NC	NC	NC	NC	NC	NC
mp-xylenes	NC	NC	NC	NC	NC	NC	NC	NC	NC	NC	NC	NC	NC
1.2.4-trimethylbenzene	NC	NC	NC	NC	NC	NC	NC	NC	NC	NC	NC	NC	NC
Naphthalene	1,24E-07	1,24E-07	1,03E-07	9,63E-08	1,22E-07	1,09E-07	2,74E-08	7,23E-08	7,75E-08	2,66E-08	4,61E-08	4,13E-08	4,50E-08
Phenol	NC	NC	NC	NC	NC	NC	NC	NC	NC	NC	NC	NC	NC
Aliphatic hydrocarbons													
n-decane	NC	NC	NC	NC	NC	NC	NC	NC	NC	NC	NC	NC	NC
n-heptane	NC	NC	NC	NC	NC	NC	NC	NC	NC	NC	NC	NC	NC
n-undecane	NC	NC	NC	NC	NC	NC	NC	NC	NC	NC	NC	NC	NC
Halogenated hydrocarbons													
1.1.1-Trichloroethane	NC	NC	NC	NC	NC	NC	NC	NC	NC	NC	NC	NC	NC
1.4-Dichlorobenzene	2,14E-07	2,19E-07	2,02E-07	1,89E-07	2,40E-07	2,14E-07	5,39E-08	1,42E-07	1,52E-07	0,00E+00	0,00E+00	0,00E+00	0,00E+00

Trichloroethylene	1,95E-08	1,99E-08	1,83E-08	1,72E-08	2,19E-08	1,94E-08	4,90E-09	1,29E-08	1,38E-08	4,75E-09	8,22E-09	7,37E-09	8,03E-09
Tetrachloroethylene	2,27E-08	2,12E-08	4,77E-09	4,47E-09	5,68E-09	5,05E-09	1,27E-09	2,55E-08	2,96E-08	1,23E-09	1,21E-09	1,92E-09	2,09E-09
Trichloromethane	5,80E-07	6,07E-07	4,22E-07	3,96E-07	5,03E-07	4,47E-07	1,13E-07	3,50E-07	3,87E-07	1,09E-07	6,96E-07	1,78E-07	1,85E-07
Dibromochloromethane	NC	NC	NC	NC	NC	NC	NC	NC	NC	NC	NC	NC	NC
Tribromomethane	2,14E-08	2,19E-08	2,02E-08	1,89E-08	2,40E-08	2,14E-08	5,39E-09	1,42E-08	1,52E-08	5,22E-09	9,05E-09	8,11E-09	8,83E-09
Alcohols													
2-ethyl-1-hexanol	NC	NC	NC	NC	NC	NC	NC	NC	NC	NC	NC	NC	NC
Ethanol	NC	NC	NC	NC	NC	NC	NC	NC	NC	NC	NC	NC	NC
Isopropanol	NC	NC	NC	NC	NC	NC	NC	NC	NC	NC	NC	NC	NC
n-propanol	NC	NC	NC	NC	NC	NC	NC	NC	NC	NC	NC	NC	NC
Ketones													
Acetone	NC	NC	NC	NC	NC	NC	NC	NC	NC	NC	NC	NC	NC
2-Butanone	NC	NC	NC	NC	NC	NC	NC	NC	NC	NC	NC	NC	NC
Terpenes													
Limonene	NC	NC	NC	NC	NC	NC	NC	NC	NC	NC	NC	NC	NC
Ethers													
Ether	NC	NC	NC	NC	NC	NC	NC	NC	NC	NC	NC	NC	NC
2-Ethoxyethanol	NC	NC	NC	NC	NC	NC	NC	NC	NC	NC	NC	NC	NC
2-Butoxyethanol	NC	NC	NC	NC	NC	NC	NC	NC	NC	NC	NC	NC	NC
Aldehydes													
Formaldehyde	**1,15E-05**	**1,13E-05**	**3,75E-05**	**1,17E-05**	**1,90E-05**	**1,21E-05**	**4,66E-06**	**1,67E-05**	**1,75E-05**	**1,67E-06**	**4,41E-06**	**2,85E-06**	**3,54E-06**
Acetaldehyde	**1,48E-06**	**1,44E-06**	**1,29E-06**	**1,02E-06**	**1,40E-06**	**1,13E-06**	3,22E-07	**1,35E-06**	**1,42E-06**	1,99E-07	8,58E-07	5,10E-07	5,00E-07
Propionaldehyde	NC	NC	NC	NC	NC	NC	NC	NC	NC	NC	NC	NC	NC
Butyraldehyde	NC	NC	NC	NC	NC	NC	NC	NC	NC	NC	NC	NC	NC
Isovaleraldehyde	NC	NC	NC	NC	NC	NC	NC	NC	NC	NC	NC	NC	NC
Valeraldehyde	NC	NC	NC	NC	NC	NC	NC	NC	NC	NC	NC	NC	NC
Hexaldehyde	NC	NC	NC	NC	NC	NC	NC	NC	NC	NC	NC	NC	NC
Phthalates													
di(2-ethyl hexyl)phthalate (DEHP)	3,40E-09	3,61E-09	6,43E-09	6,99E-09	1,26E-08	6,95E-09	3,17E-09	3,18E-09	3,54E-09	4,14E-09	4,97E-09	3,19E-09	3,42E-09
Diethylphthalate (DEP)	NC	NC	NC	NC	NC	NC	NC	NC	NC	NC	NC	NC	NC
Dibutylphthalate (DBP)	NC	NC	NC	NC	NC	NC	NC	NC	NC	NC	NC	NC	NC
Diisobutylphthalate (DiBP)	NC	NC	NC	NC	NC	NC	NC	NC	NC	NC	NC	NC	NC
Benzylbutylphthalate (BBP)	NC	NC	NC	NC	NC	NC	NC	NC	NC	NC	NC	NC	NC
Diisononylphthalate (DiNP)	NC	NC	NC	NC	NC	NC	NC	NC	NC	NC	NC	NC	NC
Musks													
Tonalide (AHTN)	NC	NC	NC	NC	NC	NC	NC	NC	NC	NC	NC	NC	NC
Galaxolide (HHCB)	NC	NC	NC	NC	NC	NC	NC	NC	NC	NC	NC	NC	NC
Pyrethrinoides													
Cyfluthrine	NC	NC	NC	NC	NC	NC	NC	NC	NC	NC	NC	NC	NC
Cypermethrine	NC	NC	NC	NC	NC	NC	NC	NC	NC	NC	NC	NC	NC
Deltamethrine	NC	NC	NC	NC	NC	NC	NC	NC	NC	NC	NC	NC	NC

Permethrine	NC	NC	NC	NC	NC	NC	NC	NC	NC	NC	NC	NC	NC
Tetramethrine	NC	NC	NC	NC	NC	NC	NC	NC	NC	NC	NC	NC	NC

Notes: Red values indicates an excess lifetime cancer risk exceeding the acceptability threshold of 1 x 10^{-5}; Orange values indicates an excess lifetime cancer risk exceeding 1 x 10^{-6}; NC indicates that the excess lifetime cancer risk exceeding was not calculated due to the absence of TRV for cancer risk.
